# Motivation toward physical activity and nutrition in older cancer patients: the MONAGE protocol using ecological momentary assessment and accelerometers

**DOI:** 10.1186/s12877-025-06130-1

**Published:** 2025-07-01

**Authors:** Mathis Brusseau, Blandine Gallet-Suchet, Gérard Dray, Sophie Gendrault, Lobna Harguem, Julie Boiché

**Affiliations:** 1 EuroMov Digital Health in Motion, Univ Montpellier, IMT Mines Alès, Montpellier, France; 2 Move in Med, Baillargues, France; 3 Department of Supportive Care, Montpellier Cancer Institute (ICM), University of Montpellier, Montpellier, France; 4 Department of Clinical Research and Innovation, Montpellier Cancer Institute (ICM), University of Montpellier, Montpellier, France

**Keywords:** Cancer, Behavior change, Exercise, Nutrition, Motivation, Accelerometers, Times series data, Ecological momentary assessment, Aging, Care pathways

## Abstract

**Background:**

Older adults with cancer struggle to maintain recommended levels of physical activity and nutrition during treatments. Collecting data in a real-life context provides a better understanding of the motivational and behavioral dynamics of this population, which is underrepresented in clinical trials. Ecological momentary assessment (EMA) is a frequently used approach to collect repeated measures in real time in a naturalistic environment. This approach can provide insights into the temporal dynamics of psychological processes, such as motivation toward health behaviors. The aim of this study is to identify clusters based on predictive motivational variables related to physical activity and nutritional behaviors among older patients with cancer, via repeated measures.

**Methods:**

In this project, older patients with a cancer (≥ 70 years old) complete physical capacity assessments, malnutrition identification, and a comprehensive geriatric assessment with G-code. After that, participants engage in a 2-week data collection protocol combining EMA and sensor-based monitoring of behavior. The participants wear an ActiGraph GT3X-BT accelerometer (ActiGraph, LLC) on their nondominant waist to measure physical activity. EMA questionnaires are delivered 3 times per day - morning (between 8am and 10am), midday (between 12pm and 2pm) and evening (between 7pm and 8pm) - via smartphones or computers with the UniQ digital application. Motivational constructs based on the Theory of Planned Behavior are collected in the morning and at midday. Nutritional behavior data are collected at midday and in the evening. Fatigue is collected in the morning, at midday and in the evening. The data analysis strategy shall include Multilevel Vector Autoregressive Models with k-means clustering.

**Discussion:**

This project seeks to identify clusters in older patients with cancer based on key motivational predictors related to physical activity and nutritional behaviors, via approaches for collecting repeated measurements in real time within a naturalistic environment. This research into the mechanisms of action between motivation and behaviors could optimize care pathways by designing appropriate intervention content, under conditions where the content will be most effective, to promote sustained behavior change among older adults with cancer.

**Trial registration:**

ClinicalTrials.gov (NCT06445140). Registered on 06 June 2024.

**Supplementary Information:**

The online version contains supplementary material available at 10.1186/s12877-025-06130-1.

## Introduction

While cancer is the leading cause of death in people aged 75 to 85, and the second leading cause of death in people over 85, there is little evidence concerning the elderly due to their under-representation in clinical trials [1]. This underrepresentation increases the risk of under or overtreatment in this population [2], making the elderly even more vulnerable to chemotherapy-related toxicities [3]. There is considerable heterogeneity in the population of patients over 70 years old. Some individuals age with few comorbidities and maintain independence, whereas others have several chronic pathologies and deficits [4]. Support for the elderly cancer patients must be comprehensive and individualized, incorporating their opinion and a multidisciplinary approach to meet all identified needs [5].

Aging leads to physiological changes (hormonal, metabolic, etc.) and an increase in risk behaviors, including physical inactivity and malnutrition [6–8]. These factors are responsible for a loss of strength and muscle mass [9], exacerbated by cancer and its treatments [10], a phenomenon that is even more pronounced in elderly individuals. This progressive and generalized loss of muscle mass and strength is associated with a deterioration in physical capacity and high rates of hospitalization and mortality [10]. In addition to muscle-related issues, these risky behaviors associated with aging also lead to the onset of other comorbidities and a higher risk of mortality [11].

In this context, nutritional support and physical activity (PA) programs / prescription are two complementary and effective interventions for maintaining muscle status and preventing malnutrition during oncology treatment. More specifically, prevention and management of this loss of muscle mass and strength should be based on adequate energy and protein intake [9, 12] and multimodal exercise (including muscle strengthening coupled with exercise conditioning) at moderate intensity [8, 9, 12–14]. These recommendations apply to both primary, age-related mass loss, and secondary, disease-related mass loss. Although an intervention combining nutritional support and regular PA practices is recognized as effective, it is difficult to achieve high adherence [13, 15], and lasting behavioral changes interventions are needed to encourage the elderly to adopt a more active lifestyle [16] and to change their eating habits [17].

Identifying the motivational variables associated with PA and nutrition in this population could facilitate the adoption and sustainability of these behaviors. Past literature indicates that using a theoretical approach to identify motivational variables associated with PA and nutrition is important to facilitate behavior change and increased adherence [18, 19]. A meta-analysis has shown that no theory is superior to another in terms of effectiveness in changing PA, and that interventions are more effective when they are based on a single theory rather than a combination of theories [18]. In this respect, the theory of planned behavior (TPB) is commonly used in behavior change studies, particularly for an aging population [19, 20]. This theory assumes that the adoption of a behavior arises from the formation of intentions in the individual, which is the most important determinant of behavior [20, 21]. These intentions are facilitated by attitudes, subjective norms (particularly perceived in the caregiver) and perceived behavioral control. Attitudes represent the benefits perceived by the characteristics of the perceived outcome expectancy, forming two distinct types of attitudes: affective attitudes, the emotion and affect-based perceptions about a behavior (e.g., dull or pleasant); and, instrumental attitudes, the perceptions related to the usefulness (e.g., worthless or valuable). Subjective norms correspond to the individual’s social influence, represented by what those around him or her think of the behavior he or she wants to undertake. Perceived behavioral control refers to the perceived ease or difficulty of performing a behavior, and thus to an individual’s belief that he or she possesses the necessary resources to perform the behavior. Moreover, this variable is assumed to be directly related to behavior. In oncology and geriatrics, intervention research indicates that an increase in TPB variables is accompanied by a significant increase in PA levels [20, 22].

However, the TPB has been criticized for its fluctuating prediction of behavioral intentions. Indeed, the designs used are often based on a limited number of measures, failing to consider the temporal fluctuation of perceptions and intraindividual variability [18]. In response to these methodological limitations, repeated-measures methods are relevant to assess the relationship between intentions and behavior on a daily basis [23, 24]. In particular, intentions can vary according to the time of day, reflecting the dynamics of motivational variables [24]. As a result, repeated-measures methods are deemed effective to estimate TPB variables and the intention-behavior gap, as they identify the contexts most conducive to a translation of intentions into action [23].

Ecological Momentary Assessment (EMA) involves repeated sampling of behaviors and experiences, which could consider the temporal fluctuation of perceptions in the subject’s natural environment [25, 26]. The EMA aims to minimize recall bias, maximize ecological validity, and allows the study of microprocesses that influence behavior in real-world contexts. This data collection method is suitable for measuring TPB variables and could improve their predictive ability. Older adults constitute a population particularly amenable to EMA research [27], with high compliance rates (around 80%) [25, 28]. Collecting repeated measures in real time within a naturalistic environment, like wearables technologies and EMA [27], is crucial for a better understanding of the behaviors for this specific population [29]. By improving the observability and feasibility of the components of an intervention, it becomes possible to develop effective, individualized interventions that consider the singularity of individuals and their situation. With the aim of developing an optimal behavior-change intervention for elderly cancer patients, further research is needed to understand the mechanisms of action on PA and nutritional behaviors.

## Materials and methods

### Study design

This is the protocol of the observational part of MONAGE, a prospective longitudinal observational cohort study, taking place over a period of 2 weeks, with a longitudinal ecological momentary assessment (EMA) phase and GT3X accelerometer worn on the waist.

The aims of the observational part of MONAGE are to identify motivational predictors related to PA and to nutritional behaviors and to observe groups of patients with similar patterns of relations between motivation and behaviors among older patients with cancer.

### Patient selection

Recruitment is anticipated to be completed by April 2026, with the aim of enrolling 40 patients in total. The patients considered for inclusion are those who come for their oncogeriatric care at the Cancer Institute of Montpellier (ICM) which is referral centre in France. If patients are eligible, the study is proposed to them by the investigator. Patients have to meet the following inclusion criteria: being aged 70 or over, having cancer (solid tumors, all sites), having a Geriatric 8 (G8) score ≤ 14, being inactive, i.e., not meeting recommended PA levels of 150 min per week at moderate intensity, providing informed, written and expressed consent and having a social security affiliation.

The exclusion criteria are as follows: known presence of brain metastases, inability to participate in digital platform or physical tests, inability to eat orally, contraindication or inability to engage in PA, inability to follow up regularly for psychological, family, numerical, social or geographical reasons and lack of liberty or under protective custody or guardianship.

This research will be conducted in accordance with the French Public Health Code, the Good Clinical Practice and the Declaration of Helsinki. This study has been registered on clinicaltrial.gov (NCT06445140). This research project has been approved by the national ethics committee (Comité des Personnes Nord Ouest III, accepted on 30 May 2024) and the “Agence nationale de sécurité du médicament et des produits de santé” (ANSM, French National Agency Authority for the Safety of Health Products) has been informed (19 June 2024). Written informed consent was obtained from all participants prior to inclusion in the study. Data will be securely stored using EasyMedStat software.

### Procedures

#### Baseline assessments and consultations

**Fig. 1:**
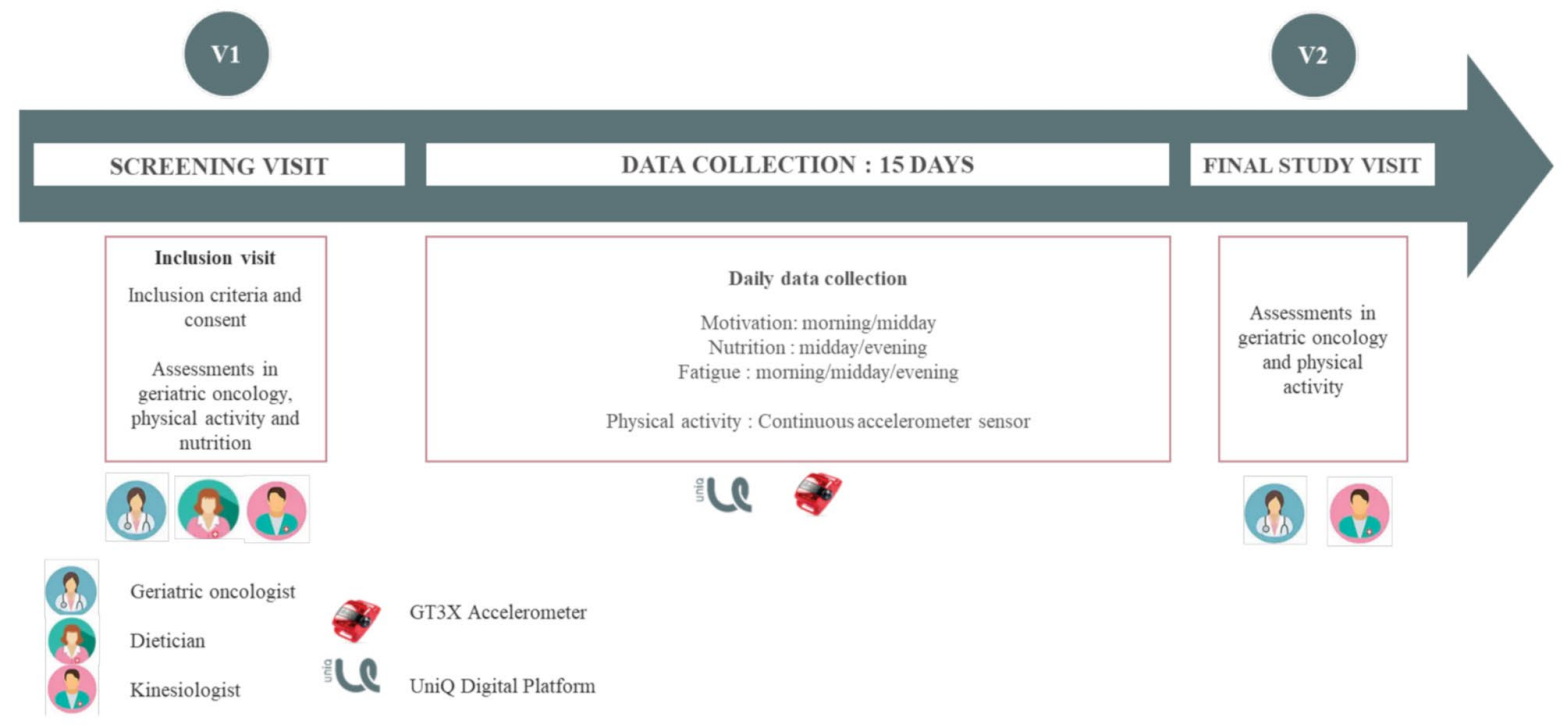
Overview of the study’s design and procedures

As part of usual care, the patient takes part in three consultations: one with a geriatric oncologist, one with a teacher in adapted physical activity (kinesiologist) and one with a dietician. Figure 1 provides an overview of the study design and assessments.

#### Oncogeriatric consultation

The patient takes part in a Comprehensive Geriatric Assessment (CGA), with a geriatric oncologist, to facilitate appropriate geriatric management. CGA is a patient-centered, multidimensional, and interdisciplinary process that results in a personalized care plan, identifying and addressing health issues that may contribute to frailty [30]. The International Society of Geriatric Oncology (SIOG) and the American Society of Clinical Oncology (ASCO) recommend incorporating geriatric assessments in the care of older patients with cancer [31]. During this consultation, the CGA includes the Geriatric Core Data Set (G-CODE) [5]. The tools/items proposed for the G-CODE are the following: (1) social assessment: living alone or support requested to stay at home; (2) functional autonomy: Activities of Daily Living (ADL; [32]) questionnaire and short Instrumental ADL questionnaire [33]; (3) mobility: Timed Up and Go test [34]; (4) nutrition: weight loss during the past 6 months and body mass index; (5) cognition: Mini-Cog test [35]; (6) mood: Mini-Geriatric Depression Scale [36] and (7) comorbidity: updated Charlson Comorbidity Index [37].

#### Exercise consultation

The patient takes part in an exercise consultation, with a teacher in adapted physical activity (kinesiologist), to evaluate PA level and capacity. The assessments proposed during the exercise consultation are the following: PA level (Global Physical Activity Questionnaire; [38]), grip strength (Handgrip with Jamar PLUS + Digital Dynamometer; [39]) and lower extremity function (Short Physical Performance Battery; [40]).

Grip strength is a clinically objective test, easy and reproducible, which measures maximum grip strength in the dominant hand by using a hand dynamometer to estimate muscle strength in the upper extremity [41]. The Short Physical Performance Battery is also an objective test designed to assess lower extremity function with three parts (Balance, Gait Speed and Chair Stands). These two objective tests are gaining increasing popularity in oncology, particularly in geriatric settings [42].

During this exercise consultation, the patient is given the Institut National du Cancer (INCa) recommendations for PA [43], which include: at least 150 min of cardiorespiratory exercise at moderate-intensity per week, at least 2 sessions per week of muscle-strengthening exercise, flexibility and mobility exercise 2 to 3 sessions per week and balance exercises at least twice a week for patients aged 65 and older. Patients are clearly informed that the concepts of individualization and progressiveness are fundamental to these recommendations. During this consultation, patients are free to ask any questions they may have. No exercise program is carried out during this consultation. However, if necessary, a prescription for the physiotherapist is given to the patient.

#### Nutritional consultation

The patient takes part in a nutritional consultation, with a dietician, to assess the nutritional status and identify needs for nutritional support, if necessary. The assessments proposed during the nutritional consultation were as follows: dietary intake (Visual/Verbal Analogue Scale of food ingesta (ingesta-VVAS); [44]) and identity nutritional risk (Mini Nutritional Assessments; [45]).

The ingesta-VVAS uses 10-point analogue scales to estimate dietary intake in patients with cancer; higher scores indicate better dietary intake and a score ≤ 7 is used as cut-off value to detect patients with nutritional risk of weight loss in medical oncology [44]. The Mini Nutritional Assessments is a validated tool to identify individuals aged 65 years or older who are at risk of malnutrition or malnourishment [46], with threshold values of ≥ 24 for well-nourished individuals, 17-23.5 for those at risk of malnutrition, and < 17 for malnourished individuals, with good validity scores [47].

During this nutritional consultation, on the basis of the European Society for Clinical Nutrition and Metabolism (ESPEN) recommendations for nutrition in geriatrics [48] and in cancer [49], the patient is given the following 3 recommendations: incorporate foods rich in protein and calories in the diet; eat small portions multiple times a day to ensure adequate intake; and drink water between meals to maintain appetite. During this consultation, patients are free to ask any questions they may have. If necessary, a prescription for oral nutritional supplements is given to the patient.

#### Preparation for data collection

At the end of the 3 consultations, if the selection criteria are validated by the geriatric oncologist and by the kinesiologist for the level of PA, the study is fully explained by the investigator. Examples of data collection questions and applications are shown to the patient on the phone and/or computer.

If the patient accepts and signs the consent form directly, a personal account is created on the digital platform “UniQ”. If the patient decides to use it on his / her phone, the investigator installs the application, adds it to the home screen, and makes the first connection with the patient. If Uniq is used on a computer, an account is created, and an email is sent to the patient to activate the account from his / her computer. A phone number is also provided to patients for support in case of technical issues.

### Outcomes measures

Data collection begins the day after inclusion and lasts for 14 days. The data are collected via digital scales on the UniQ application via an EMA approach and a GT3X accelerometer worn on the waist. To observe whether the protocol seemed feasible for patients during the creation of the intervention, 7 patients with similar profile to those likely to be included were questioned about the frequency and duration of data collection. The retained configuration (i.e., answering to 3 very short questionnaires per day, and wearing the accelerometer for 14 days) suited both the patients and the investigators. The estimated time taken to complete the 3 questionnaires for a patient is 10 min per day, i.e. 4 min for the morning and midday questionnaires (12 items) and 2 min for the evening questionnaire (2 items). Every time a questionnaire is sent, patients receive an automatic e-mail informing them that a questionnaire is available on their UniQ platform and inviting them to answer. If necessary, the patient is informed that the caregiver can help him to complete the questionnaires.

#### Motivation toward physical activity and nutrition

Twice a day patients are asked about their motivation toward PA and nutrition over the next few hours: in the morning (between 8am to 10am) and at midday (between 12pm and 2pm). For each behavior, the questionnaire comprised five items corresponding to the TPB and assessing the following constructs: attitudes, both affective (e.g., “In my opinion, doing physical activity for at least 10 minutes in the next few hours would be a source of pleasure”) and instrumental (e.g., “For me, engaging in physical activity for at least 10 minutes in the next few hours would be beneficial for my health”); subjective norms, referring to perceived social approval from family members, significant others, or health professionals (e.g., “For me, doing physical activity for at least 10 minutes in the next few hours would be approved by most of the important people in my life”); perceived behavioral control (e.g., “It would be easy for me to do physical activity for at least 10 minutes in the next few hours”); and intention (e.g., “I intend to engage in physical activity for at least 10 minutes in the next few hours”). Each item uses a 7-point numeric scale to estimate motivation toward the behavior, with threshold values: 1 Not at all – 3 A bit – 5 Quite – 7 Very much. These questions were created following the guide to prepare a TPB questionnaire [50]. To validate these questionnaires, 7 patients with similar profiles to those likely to be included were asked about their understanding of the questions. To avoid repetitions during the study, 5 different versions were created and alternatively presented (see the complete list in the supplementary files).

#### Fatigue

Patients are asked about their fatigue 3 times a day: in the morning (between 8am and 10am), at midday (between 12pm and 2pm) and in the evening (between 6pm to 8pm). The patient answer thanks to the item: “At the moment, I feel tired” with a 7-point numeric scale with threshold values: 1 Not at all – 3 A bit – 5 Quite – 7 Very much.

#### Nutrition

Patients are asked about their dietary intake twice a day: at midday (between 12pm and 2pm) and in the evening (between 6pm and 8pm). The patient are asked: “If you consider that when you are healthy you eat at 10/10, today you ate at?”, and answer through a 10-point numeric scale with: 1 I don’t eat anything – 10 I eat as usual.

#### Physical activity

The PA data are measured via an accelerometer (ActiGraph GT3X-BT), which provides a reliable and valid objective measure [51]. Patients are instructed to wear the device on their nondominant waist all day for the entire data collection period, except during the sleep period. The ActiGraph GT3X-BT accelerometer provides a device-based measure of PA. If patients engage in activities where the monitor could get wet (e.g., swimming, showering or bathing), they are instructed to remove it. The data are recorded in 30-second epochs. Two day-segments were defined: midnight to 1pm for morning PA and 1pm to midnight for afternoon PA.

The PA intensity is graded based on the Euclidean Norm Minus One (ENMO). The objective is to indicate the threshold intensity based on ENMO to separate inactivity from light, light from moderate, and moderate from vigorous PA, respectively. We will use the following cutoff points: 14 for light (threshold.lig) and 70 for moderate (threshold.mod) intensity PA. These cutoff points are based on two studies among older adults: Sanders et al., in a sample of participants aged from 60 to 86 years old, set the cutoff points at 15 for threshold.lig and 69 for threshold.mod [52], and Migueles et al., among participants older than 70 years old (mean equal to 78.7 years old) defined cutoff points as 18 for threshold.lig and 60 for threshold.mod [53].

### Preprocessing data

To analyze the data of ActiGraph GT3X-BT, we will use the GGIR R-package [54]. This package is useful for quantifying the PA with precision by processing multiday raw accelerometer data. The term raw refers to data expressed in m/s^2^ or gravitational acceleration. We use the “qwindow” argument of GGIR to specify whether and how to segment the day for day-segment specific analysis.

Missing data will be imputed via the Kalman filter for both the accelerometer and EMA data. The Kalman filter is a dynamic imputation method that estimates missing values in repeated measures by using both past and present information, accounting for temporal patterns and measurement noise to produce plausible and consistent data over time. The Kalman filter is a popular method for data imputation in time series data, especially those recorded by wearable technology [55] and in EMA conditions [56]. Depending on the characteristics of the dataset, a threshold for acceptable missing data will be established to retain as many valid cases as possible without compromising data quality and to ensure sufficient temporal information per individual. Although the Kalman filter can impute missing values under the assumption of temporal continuity, a minimum amount of observed data is necessary to reliably capture intra-individual dynamics [57].

### Statistical analysis

The objective of our study is to identify clusters based on predictive motivational variables related to PA and nutritional behaviors among older patients with cancer. The analyses to be conducted aim (i) to identify predictors of PA and nutritional behaviors and (ii) to identify groups of patients with similar patterns of relations between motivation and behavior.

We will evaluate the sample’s adherence to and acceptance of this original data collection with descriptive statistics such as the means, medians, and percentages. These measures will allow us to summarize the key characteristics of the dataset.

For the first step, we will use a Multilevel Vector-autoregressive model (mlVAR), as implemented in the R package “mlVAR.”. mlVAR model will be run in parallel for physical activity and nutrition separately. The mlVAR is a multilevel extension of the vector auto-regression (VAR) model, which describes dynamic relationships among variables measured repeatedly over time from multiple subjects [58]. This model has become a popular tool particularly in studies involving psychological variables [59]. In a contemporaneous network, mlVAR identifies relationships between variables over the same time interval. To observe the temporal dynamics between variables in mlVAR, lagged coefficients quantify the auto-regressive effect (one variable on itself) and cross-lagged effect (one variable on another variable). In this temporal network, these coefficients illustrate the predictive relationships between variables over time and identify potential causal relationships. As a hypothetical example, we can observe that intention at time *t* predicts behavior at time *t + 1*, suggesting a directional influence of intention on subsequent behavior over time.

In a second step, we will use clustering methods (e.g., k-means) based on the coefficients of the VAR model, to identify groups of participants with similar patterns of relationships between motivation and behavior (nutrition and PA). K-means analysis will be run in parallel for physical activity and nutrition separately. Silhouette coefficients will be used to evaluate the validity and stability of the clustering [60], by comparing how close a point is to its own cluster, which is equivalent of the similarities among all individuals in the same cluster, versus how far it is from the nearest neighboring cluster. Its values range from − 1 to 1, where 1 indicates good clustering and − 1 indicates inaccurate clustering. The Silhouette coefficients are particularly relevant, because no additional training data is required [61].

### Creation of an intervention

The results of the observational part of MONAGE will support the design of an effective and optimized behavior-change intervention for elderly cancer patients, thanks to a tailored approach of the relations between motivation and behaviors. The goal of the interventional part of MONAGE will be to improve physical activity and nutritional behaviors in a new sample of participants. This intervention will be designed in collaboration with patients, their caregivers / partners, doctors, dieticians, adapted physical activity teachers (kinesiologists), and behavior change professionals. Intervention content will be targeted on the most relevant strategies, based on the results in the observational phase; in other words, the choice and use of behavior change technique [62, 63], which are known to significantly lead to a change of PA behavior [64] and nutrition [65]. For example, “13.2. Framing/reframing”, a behavior change technique which is known to significantly influence attitudes toward the behavior [66], will only be proposed to participants for whom attitudes toward the behavior attitudes have emerged as a significant predictor of the behavior. For this purpose, during the interventional phase of MONAGE, each patient will undergo a one-week data collection period, similar to that of the observational phase, to determine whether they belong to one of the clusters identified during the observational phase. This identification phase then allows for the delivery of the intervention tailored to the patient’s profile.

## Discussion

### Expects findings

This study aims to identify clusters based on predictive motivational variables related to PA and nutritional behaviors among older patients with cancer, via repeated measures. First, sufficient patient participation and adherence to the protocol are expected, with a good compliance completion rate for EMA and Actigraph GT3X-BT wear. Compliance was considered good if at least 70% of the planned EMA measures were completed across the study period, with fewer than 30% of missed responses. Participants were considered non-compliant if they responded to less than 50% of prompts. Participation and compliance will enhance our knowledge about how EMAs and accelerometers can be used among older adults during cancer treatment. With mlVAR analysis, we will attempt to identify predictors of behaviors based on motivational variables. Second, the analyses should identify groups of patients with similar patterns of relationships between motivation and behavior, by using clustering methods (e.g. k-means) based on the coefficients of the mlVAR model.

### Strengths and limitations

There are several limitations in the design of our study that need to be considered. First, we acknowledge that the selection criteria do not allow a complete representation of the elderly cancer population. Active patients and those who were not sufficiently connected were not included in the study. Daily nutrition data collection is not a validated measure, and is only based on the ingesta-VVAS, which may limit validity. The use of the mlVAR is particularly interesting for analysis, but they cannot capture how variables evolve outside predefined measurement occasions in our study (e.g. fatigue or motivation) [67]. The number of patients and the length of the data series may also limit analysis, so preliminary tests (silhouette coefficient for clustering; [60]) and adjustments to recruitment may be carried out.

One of the key strengths of this study is the recruitment of elderly cancer patients (≥ 70 years old), who are particularly underrepresented in clinical research [4]. We will carry out a complete assessment of our recruited population including a CGA, G8 [68] and G-CODE score, grip strength and SPPB assessment [42] and dietary intake, which will provide a complete profile of the patients included. The data collection is based on repeated sampling of behaviors and reflective motivational processes, in real time, which could consider the temporal fluctuation of perceptions in the individual’s natural environment. For this purpose, EMA and wearable devices are frequently used among clinical researchers to collect naturalistic data in real time [28, 67]. EMA questionnaires were designed with patients, and patient feedback was given on the UniQ digital application to promote good feasibility, which corresponds to the health literacy and digital literacy of the elderly [69].

### Future directions

Studying the relationship between motivation and behaviors in real-time can provide additional important insights into the psychological states of PA and nutrition in older patients with cancer. Exploring this relationship to observe predictors of behaviors on a daily or within-daily basis is another exciting avenue for future research toward behavior change and understanding the variation in daily behaviors among older patients with cancer. Future individualized interventions can build on these results to increase patient compliance with physical activity and nutrition interventions. In summary, this research into understanding the mechanisms of action between motivation and behaviors could optimize care pathways by designing appropriate intervention content, under conditions where the content will be most effective, to promote sustained behavior change among older adults with cancer in PA and nutrition. For this, a behavior-change intervention will be designed in the interventional part of MONAGE to improve physical activity and nutritional behaviors in a new sample of elderly cancer patients.

## Electronic supplementary material

Below is the link to the electronic supplementary material.

Supplementary Material 1

Supplementary Material 2

## Data Availability

No datasets were generated or analysed during the current study.

## Abbreviations

ADL: Activity of Daily Living
ASCO: American Society of Clinical Oncology
CGA: Comprehensive Geriatric Assessment
EMA: Ecological Momentary Assessment
ENMO: Euclidean Norm Minus One
ESPEN: European Society for Clinical Nutrition and Metabolism
G-CODE: Geriatric Core Data set
IADL: Instrumental Activity of Daily Living
INCa: Institut National du Cancer
Ingesta-VVAS: Ingesta Visual Verbal Analogue Scale
mlVAR: Multilevel Vector Autoregressive Model
PA: Physical Activity
SIOG: International Society of Geriatric Oncology
TPB: Theory of Planned Behavior
VAR: Vector Autoregressive Model
