# Supplementary material for: Motivation toward physical activity and nutrition in older cancer patients: the MONAGE protocol using ecological momentary assessment and accelerometers
Source: BMC Geriatr. 2025 Jul 1;25:431. doi: 10.1186/s12877-025-06130-1 (PMC12220263; doi:10.1186/s12877-025-06130-1)
Supplement: Supplementary file 1 — Supplementary Material 1 [file 12877_2025_6130_MOESM1_ESM.docx]

**The 5 versions of the questionnaire for TPB constructs**

**Physical Activity**

**Version 1**

- Engaging in physical activity for at least 10 minutes in the next few hours could help me manage my stress.
- In my opinion, doing physical activity for at least 10 minutes in the next few hours would be enjoyable.
- For me, doing physical activity for at least 10 minutes in the next few hours would be approved by most of the important people in my life.
- I feel capable of being physically active for at least 10 minutes in the next few hours.
- I am going to engage in physical activity for at least 10 minutes in the next few hours.

**Version 2**

- For me, engaging in physical activity for at least 10 minutes in the next few hours would be beneficial for my health.
- In my opinion, doing physical activity for at least 10 minutes in the next few hours would be a source of pleasure.
- My close circle might encourage me to engage in physical activity for at least 10 minutes in the next few hours.
- It would be easy for me to do physical activity for at least 10 minutes in the next few hours.
- I intend to engage in physical activity for at least 10 minutes in the next few hours.

**Version 3**

- Doing physical activity for at least 10 minutes in the next few hours could help me manage my fatigue.
- For me, doing physical activity for at least 10 minutes in the next few hours could bring a sense of pleasure.
- My doctor would approve of me doing physical activity for at least 10 minutes in the next few hours.
- I think I can do physical activity for at least 10 minutes in the next few hours.
- I have planned to engage in physical activity for at least 10 minutes in the next few hours.

**Version 4**

- Engaging in physical activity for at least 10 minutes in the next few hours would be beneficial for my health.
- For me, doing physical activity for at least 10 minutes in the next few hours would be pleasant.
- In my opinion, my family members would want me to engage in physical activity for at least 10 minutes in the next few hours.
- Despite fatigue, I feel that I can do physical activity for at least 10 minutes in the next few hours.
- I plan to engage in physical activity for at least 10 minutes in the next few hours.

**Version 5**

- In my opinion, doing physical activity for at least 10 minutes in the next few hours would be good for my health.
- For me, engaging in physical activity for at least 10 minutes in the next few hours could be an enjoyable experience.
- I believe my close circle would accept me engaging in physical activity for at least 10 minutes in the next few hours.
- I have the ability to do physical activity for at least 10 minutes in the next few hours.
- I estimate that my chances of doing physical activity for at least 10 minutes in the next few hours are high.

**Nutrition**

**Version 1**

- Eating a sufficient amount at my next meal would be beneficial for my health (>8/10 on the intake scale).
- In my opinion, my next meal will be enjoyable.
- Eating a sufficient amount at my next meal would be approved by most of the important people in my life (>8/10 on the intake scale).
- I have the ability to eat a sufficient amount at my next meal, even if I don't enjoy the taste (>8/10 on the intake scale).
- I intend to include a sufficient amount at my next meal (>8/10 on the intake scale).

**Version 2**

- In my opinion, the quantity of my next meal is an important factor for my well-being.
- In my opinion, my next meal will be pleasant.
- My close circle would encourage me to eat a sufficient amount at my next meal (>8/10 on the intake scale).
- I feel capable of eating a sufficient amount at my next meal (>8/10 on the intake scale).
- I estimate that my chances of eating a sufficient amount at my next meal are high (>8/10 on the intake scale).

**Version 3**

- Eating a sufficient amount at my next meal is important to manage my fatigue (>8/10 on the intake scale).
- I will enjoy eating my next meal.
- My doctor would encourage me to eat a sufficient amount at my next meal (>8/10 on the intake scale).
- It will be easy for me to eat a sufficient amount at my next meal (>8/10 on the intake scale).
- I will eat a sufficient amount at my next meal (>8/10 on the intake scale).

**Version 4**

- Eating a sufficient amount at my next meal would be helpful to stay in good shape (>8/10 on the intake scale).
- Eating my next meal will be a source of pleasure for me.
- In my opinion, my family members would want me to eat a sufficient amount at my next meal (>8/10 on the intake scale).
- I am capable of making the effort to eat a sufficient amount at my next meal, even if I’m not hungry (>8/10 on the intake scale).
- I intend to eat a sufficient amount at my next meal (>8/10 on the intake scale).

**Version 5**

- It is important that I eat a sufficient amount at my next meal, even if I am not hungry (>8/10 on the intake scale).
- In my opinion, my next meal could bring me a sense of pleasure.
- My close circle would encourage me to eat a sufficient amount at my next meal (>8/10 on the intake scale).
- I can eat a sufficient amount at my next meal without difficulty (>8/10 on the intake scale).
- I plan to make the effort to eat a sufficient amount at my next meal (>8/10 on the intake scale).
